# Cancer/Testis Antigen MAGE-C1/CT7: New Target for Multiple Myeloma Therapy

**DOI:** 10.1155/2012/257695

**Published:** 2012-03-11

**Authors:** Fabricio de Carvalho, André L. Vettore, Gisele W. B. Colleoni

**Affiliations:** ^1^Disciplina de Hematologia e Hemoterapia, Universidade Federal de São Paulo, UNIFESP/EPM, Rua Botucatu, 04023-900 Vila Clementino, SP, Brazil; ^2^Departamento de Ciências Biológicas, UNIFESP, 09972-270, Diadema, SP, Brazil

## Abstract

Cancer/Testis Antigens (CTAs) are a promising class of tumor antigens that have a limited expression in somatic tissues (testis, ovary, fetal, and placental cells). Aberrant expression of CTAs in cancer cells may lead to abnormal chromosome segregation and aneuploidy. CTAs are regulated by epigenetic mechanisms (DNA methylation and acetylation of histones) and are attractive targets for immunotherapy in cancer because the gonads are immune privileged organs and anti-CTA immune response can be tumor-specific. Multiple myeloma (MM) is an incurable hematological malignancy, and several CTAs have been detected in many MM cell lines and patients. Among CTAs expressed in MM we must highlight the * MAGE-C1/CT7* located on the X chromosome and expressed specificity in the malignant plasma cells. MAGE-C1/CT7 seems to be related to disease progression and functional studies suggests that this CTA might play a role in cell cycle and mainly in survival of malignant plasma cells, protecting myeloma cells against spontaneous as well as drug-induced apoptosis.

## 1. Cancer/Testis Antigens

Tumors generally are immunogenic. They produce proteins that normally are not expressed in tissues from adults and therefore are not considered self by the immune system [1]. 

The idea that immune system can recognize and respond to these tumor proteins (antigens) was postulated at the end of the 19th century, when William Coley, a surgeon at Memorial Sloan-Kettering Cancer Center in New York (USA), observed that rare events of spontaneous tumor progression were often preceded by infectious episodes [2].

Tumor immunology began several decades ago, when it was shown that mice could be immunized against syngeneic tumors and that the antibodies produced led to specific rejection of transplanted tumor tissue [3, 4]. Thomas and Burnet introduced the concept of cancer immune surveillance to describe a mechanism of protection against tumors in immunocompetent hosts. From this, the development of tumor vaccines for the human population became a new possibility for cancer treatment [2].

Tumor-associated antigens were originally discovered in patients with malignant melanoma. These antigens were subsequently identified in several types of human tumors. In normal tissue, they were first described in testicular germ line. Therefore, genes that express these proteins were called *cancer/testis antigens* (CTAs) [5–7].

Thus, the CTAs are a promising class of tumor antigens due to its limited expression in somatic tissues (germ cells of the testis, ovary, fetal, and placental cells (trophoblast)) [8–10]. Some CTAs can be expressed in other normal tissues such as pancreas, liver, and spleen, but the level of expression is much smaller than observed in germ cells [9].

The study by van der Bruggen et al. [11] was the first to demonstrate that CTAs could be specific recognized *in vitro *by cytotoxic T lymphocytes (CTLs) in patients with melanoma. It was also possible to obtain autologous antitumor CTL by cultures of irradiated tumor cells with blood lymphocytes of melanoma-bearing patient [12, 13].

Testicular and placental cells did not express MHC (*Major Histocompatibility Complex*) class I, and the CTAs are not recognized by CD8+ cytotoxic T lymphocytes. For this reason, CTAs are considered excellent targets for antitumor cell vaccines [14].

The development of vaccines for specific-tumor antigens depends in part on the identification of a broad spectrum of immunogenic proteins expressed predominantly in human cancer. The technique of cloning T-cell epitopes, developed and published in 1991, led to the discovery of the human CTAs MAGEA1, BAGE, and GAGE. It was demonstrated that the products of the mRNAs transcribed and translated these CTAs almost exclusively in normal testis and various tumor types [15].

In 1995, the SEREX (*Serological Analysis of cDNA Expression Libraries*) technique was used in the search of new tumor antigens recognized by IgG of cancer patients [16]. Among the genes identified by SEREX in human tumors were *SSX2*, *NY-ESO-1,* and *SYCP-1*, which were also, predominantly, expressed in normal testis and cancer [15].

Scanlan et al. [15] classify the CTAs into four categories, according to the expression profile measured qualitatively by conventional RT-PCR (*Reverse Transcriptase-Polymerase Chain Reaction*): (1) CTAs exclusively expressed in testis and tumors (*Testis-Restricted*), (2) CTAs expressed in two or more nongametogenic tissues (*Tissue-Restricted*), (3) CTAs expressed in 3–6 nongametogenic tissues (*Differentially Expressed*), and (4) CTAs expressed in >6 nongametogenic tissues (*Ubiquitously Expressed*) [15, 17, 18].

With the description of an increasing number of CTAs, it was necessary to implement a nomenclature to differentiate these genes. In general, due to lack of information about the CTA functions in cellular environment, the nomenclature was based on the chronological order of discovery (e.g., MAGEA is CT1; BAGE is CT2). There are cases of multiple members in CTA families; in those cases, each member of a family is assigned as a number, for example, SSX1 is CT5.1, SSX2 is CT5.2, SSX3 is CT5.3, and so on [15].

In 2008, a new classification of CTAs was developed from *in silico* analysis of cDNA Database (http://evocontology.org), MPSS (*Massively Parallel Signature Sequencing*), CAGE (*Cap Analysis Gene-Expression*) and data expression obtained by conventional RT-PCR. In this classification, the CTAs were divided basically into three distinct groups: *Testis-Restricted* (CTAs expressed in normal adult testis and placenta), *Testis/Brain-Restricted* (CTAs expressed in normal adult testis and in all brain tissues), and *Testis-Selective* (CTAs were classified according to the ratio between the expression of normal adult testis/placenta in relation to others expressed in normal adult tissues) [6].

**Figure 1:**
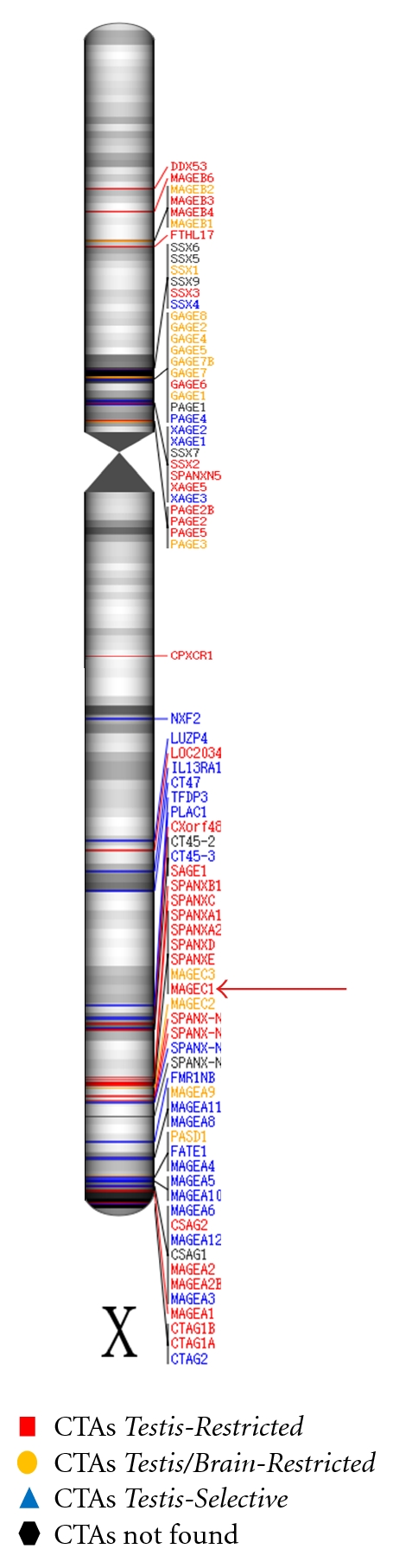
Distribution of cancer/testis antigens (CTAs) on the X chromosome. Black regions on chromosome demonstrate a high density of ESTs (*Expressed Sequence Tags*). Red Arrow indicates CTA MAGE-C1/CT7 (*Testis-Restricted*) (Font: Hofmann et al. [6]; modified by de Carvalho et al. 2011).

More than 250 CTAs were described in the CT antigen database (http://www.cta.lncc.br). The CTAs can be divided between those who are located on the X chromosome and those which are present in other chromosomes (the autosomes) [19, 20]. CTAs located on the X chromosome, such as *MAGE-C1/CT7* gene, tend to form families that are normally expressed in spermatogonia in a coordinated manner [21, 22] (Figure 1). The careful annotation of the genes present on the X chromosome showed that approximately 10% of them are CTAs and are often coexpressed in tumor cells [19, 22, 23].

In the testis, CTAs are typically expressed in spermatocytes and act in meiosis. Thus, the aberrant expression of CTAs in cancer cells may lead to abnormal chromosome segregation and aneuploidy, justifying its importance in tumorigenesis [22, 24].

CTAs expression is regulated by epigenetic mechanisms such as hypermethylation of the promoter region of the genes (DNA methylation) and acetylation of histones [9, 19, 20, 22]. Therefore, since CTAs are not expressed or have low expression in differentiated somatic tissues, some authors suggest that the expression of CTAs in tumor tissue may be restricted to cells that retain stem cell properties [9, 25]. In tumors, a restricted population with stem cell properties (cancer stem cells) can favor tumor maintenance, proliferation, and metastasis [9].

The *MAGE* genes may be involved in tumor transformation or in some aspects of tumor progression such as in tumor metastasis [11]. The *MAGE* genes are frequently expressed in human tumors of different histological types but not expressed in normal tissues except in male germ cells. The CTAs encoded by MAGE genes are recognized by CTL and are strictly tumor-specific [13].

Thus, all CTAs are in principle attractive targets for immunotherapy in cancer because the gonads are immune privileged organs and anti-CTA immune response can be tumor-specific. Vaccines using peptides derived from NY-ESO-1 (CTAG-1B) have shown clinical benefits in patients with melanoma [26, 27].

## 2. CTAs Expression in Multiple Myeloma

Multiple myeloma (MM) is a hematological malignancy secondary to clonal expansion of plasma cells, characterized by the presence of monoclonal immunoglobulin in blood and/or urine, lytic bone lesions, and infiltration of monoclonal plasma cells in bone marrow [28–30].

MM corresponds to 1% of all malignancies and 10–15% of hematologic malignancies and it is the second most common type of blood cancer [14, 31–34]. In the United States, 20,000 new cases of MM are diagnosed every year, with about 11,000 deaths by this disease in the same period of time [33, 35].

The diagnosis of MM is based on the presence of monoclonal protein (M protein) in serum and/or urine, bone marrow infiltration by at least 10% of clonal plasma cells, and damage to one or more target organs (CRAB: hypercalcemia, renal failure, anemia, bone lesions). Individuals with multiple myeloma must be distinguished from those with *Monoclonal Gammopathy of Undetermined Significance* (MGUS) (<10% plasma cells in bone marrow, low levels of M-protein (<3 g/dL), and no osteolytic lesion), amyloidosis, or other lymphoproliferative disorders with paraproteinemia [36].

The characterization of the mechanisms responsible for the expansion of MM tumor cells is difficult and involves a series of genetic alterations and changes in the bone marrow microenvironment, promoting tumor growth and the failure of the immune system to recognize it [28, 36].

Regardless of prognostic factors, MM remains incurable with median overall survival of 3–5 years [32, 35, 37–40]. Although it is possible to obtain complete remission of disease in approximately 25–50% of patients with initial diagnosis (treated with high-dose melphalan and autologous hematopoietic stem cell transplant), almost all will relapse within 2-3 years [18], suggesting that an effective maintenance therapy is needed to control or slow the progression of the disease [37, 41].

Evidences suggest that small fractions of MM cells escape the action of chemotherapy and remains undetectable by conventional methods, explaining the recurrence of the disease. These small fractions of myeloma cells are considered potential targets for immunotherapy, either active or passive, due to two main factors: (1) small fractions of MM cells can be destroyed by cytotoxic T lymphocytes, and (2) in allogeneic hematopoietic stem cell transplants, infusion of donor T cells can efficiently eliminate the small fraction of residual MM cells [18].

The vaccines formulated with antigens associated with MM can instruct the immune system to eliminate malignant cells. But specific antigens of myeloma cells are required [14].

The immunoglobulin (Ig) clones that are produced by small fractions of residual MM cells are considered ideal targets for building and specific anti-idiotype vaccines. However, several clinical studies using anti-idiotype vaccines targeting the Ig failed to demonstrate benefit in patients with MM [18].

CTAs have been detected in many cell lines and primary tumor samples from patients with MM by RT-PCR and immunohistochemistry [14]. Despite the little information available about their importance as clinical prognostic factors or related to aberrant proliferation of malignant plasma cells, some studies with different tumor cell lines have shown an association between the expression of CTAs and a phenotype of resistance to chemotherapy treatments [42, 43].

There are evidences that CTAs are also expressed in relapsed MM samples and may be considered important prognostic markers in newly diagnosed MM patients and in relapsed cases [44]. On the other hand, the expression of CTAs in many hematologic malignancies such as leukemia and B-cell non-Hodgkin's lymphomas is considered a rare event [45].

The expression of CTA members of the MAGE family in tumor cells appears to contribute directly to the malignant phenotype and poor response to therapy [22, 46, 47]. MAGE family members are present on the X chromosome and are described as CTA-X-MAGE. Moreover, all members of this family present a 200 amino acids common domain, known as MHD (MAGE *Homology Domain*), involved in protein-protein interactions [48, 49].

In our previous study, Andrade et al. [50] showed that three CTAs (localized on the X chromosome) MAGE-C1/CT7, MAGE-A3/6, and LAGE-1 were often expressed in MM suggesting that they could be good candidates for immunotherapy. According this study, CTA *MAGE-C1/CT7* gene was the most frequently expressed CTA in MM and seems to have prognostic impact in overall survival.

## 3. MAGE-C1/CT7: New Target for Immunotherapy in Myeloma

Tumor-specific immunotherapy is a promising strategy for treating patients with MM, but a T-cell-based therapy depends on identification of an antigen expressed strictly in tumor cells. Myeloma is a tumor of B cells and therefore has the potential to present antigen directly to T cells, although the ability to present the malignant plasma cells is believed to be limited. The antigen presentation of CTA proteins can thus arise from cross-priming by dendritic cells. Due to MAGE-C1/CT7 antigen expression in restricted in tumor cells, it seems to be a promising candidate for immunotherapy in MM [51].

The *CT7* gene is located in the region Xq26-27 (Figure 1) and was identified by SEREX in the melanoma cell line SK-MEL-37 and allogeneic serum of melanoma patients [52]. The *CT7* gene is identical to *MAGE-C1*, identified by RDA (*Representational Difference Analysis*) [53, 54]. Near the region of *MAGE-C1/CT7* are two subfamilies that are MAGE-B (composed of four genes that are located in the region Xq21.3) and MAGE-A (with 12 members located in the region Xq28). MAGE-C1/CT7 represents the first member of a new subfamily [53].

**Figure 2:**
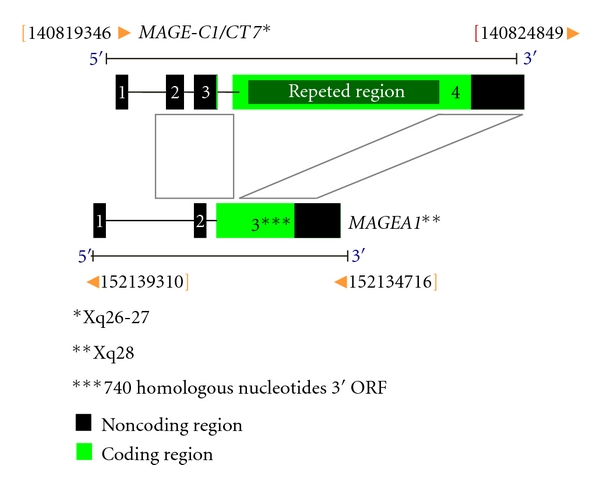
Schematic representation of MAGE-C1/CT7 gene: comparison of content structure *MAGE-C1/CT7* and *MAGEA1* genes. The ORFs (*Open Reading Frames*) are indicated in green. The black regions are exons. Repeated region of the *MAGE-C1/CT7* gene is highlighted in exon 4. The gray areas show regions of homology between the two CTAs (Font: Lucas et al. [53]; modified by de Carvalho et al. 2011).

In evolutionary terms, *MAGE-C1/CT7* gene is considered recent, present in primates (chimpanzee and *rhesus*) and humans. MAGE-C1/CT7 protein has a region homologous to the MAGE family, corresponding to 275 amino acids of the carboxyl-terminal region. In the amino-terminal region, a segment composed of tandem repeats presents several SNPs (*Single Nucleotide Polymorphisms*) (Figure 2). The most common amino acids in this repeated region are serine, proline, and glutamine, representing 53% of the whole sequence. MAGE-C1/CT7 is unique compared to MAGE families' proteins because the repeated region of this CTA has a distinct conformation compared to the other MAGE proteins [52, 53]. Lucas et al. [53] demonstrated that the repeated region presented in the amino-terminal of MAGE-C1/CT7 protein ranged from 667 to 1052 amino acids, resulting in the identification of six distinct alleles.

MAGE-C1/CT7 is considered a testis-restricted CTA by the current classification [6], because it is only expressed in normal adult testis and several studies have shown that this CTA is expressed in a wide variety of human tumors [6, 53, 55].

Cho et al. [19] demonstrated that MAGE-C1/CT7 protein is preferably located in the cytoplasm, but it has also been found in the cell nucleus. The same authors suggested that there is physical interaction between MAGE-C1/CT7 and NY-ESO-1 proteins, suggesting that the coordinated expression of two genes is a common event in many types of tumors, including MM [19]. Moreover, the expression of *MAGE-C1/CT7* in MM is seen as being restricted to the malignant plasma cells [14, 34].

Dhodapkar et al. [56] demonstrated that MAGE-C1/CT7 protein was expressed in most samples from MM, medullary plasmacytoma, and extramedullary plasmacytoma by immunohistochemistry. The same authors also observed the expression of MAGE-C1/CT7 antigen on the cell surface of the CAG cell line by flow cytometry and one case of plasmacytoma by immunohistochemistry, suggesting the expression of this CTA on the cell surface myeloma. However this fact should be future investigated because it was the only study to detect the expression of CTA on the cell surface [56]. 

Jungbluth et al. [14] observed that *MAGE-C1/CT7* gene expression is related to disease progression in myeloma due to its high expression in samples of MM stage III, compared to individuals with MGUS. Condomines et al. [18] demonstrated that MAGE-C1/CT7 was more expressed (66%) in patients with newly diagnosed MM and in those who survived the treatment. Tinguely et al. [57] showed that the expression of *MAGE-C1/CT7* does not correlated with survival of malignant plasma cells but observed that patients with MAGE-C1/CT7 protein located in the cell cytoplasm had a better prognosis than those patients who showed protein expression in the nucleus.

Andrade et al. [50] observed high-frequency (77%) *MAGE-C1/CT7* expression in MM patients with advanced stage and possible unfavorable impact on prognosis related to expression of this gene. In the same study, the *MAGE-C1/CT7* expression was also observed in patients with MGUS (33%) and bone marrow of patients with solitary plasmacytoma (20%).

Curioni-Fontecedro et al. [58], through an immunogenicity study of MAGE-C1/CT7 antigen *in vivo,* showed that this CTA was responsible for the high frequency of specific IgG antibodies in MM patients. Furthermore, they observed specific immune response against the MAGE-C1/CT7, demonstrating that antimyeloma immunity can be generated in patients with this disease.

Atanackovic et al. [59], evaluating the prognostic value of *MAGE-C1/CT7* expression in MM, demonstrated that patients undergoing allogeneic hematopoietic stem cells transplant showed early recurrence and worse overall survival when the malignant plasma cells from bone marrow expressed *MAGE-C1/CT7*. The same authors suggest that the CTAs in general are involved in the progression of myeloma, further increasing the aggressiveness of the tumor and that the *MAGE-C1/CT7* might be considered as a *gatekeeper* gene for other CTAs [59].

Nuber et al. [55] identified and characterized naturally occurring MAGE-C1/CT7-specific T lymphocytes in patients with melanoma expressing this CTA, suggesting a strong immunogenicity of this antigen and that MAGE-C1/CT7 could be a good candidate for immunotherapy. Lendvai et al. [60] demonstrated the presence of T lymphocytes specific for MAGE-C1/CT7 in patients with MM.

Anderson et al. [61] identified immunogenic CD8+ T-cell epitopes of MAGE-C1/CT7 and demonstrated that these epitopes are naturally processed and presented by tumor cells.

Atanackovic et al. [45] throughout transient silencing of MAGE-C1/CT7 and MAGE-A3 genes suggested that both CTAs are involved in the survival of myeloma cells, decreasing apoptosis induced by chemotherapy. The same authors also demonstrated that transient silencing of *MAGE-C1/CT7* in MM cell lines affected the *MAGE-C2/CT10* expression, indicating a possible interaction between both genes [45].

Recently, our group (de Carvalho et al.) [24] throughout stable silencing of* MAGE-C1/CT7 *by shRNA (*Short Hairpin RNA*) in MM cell lines showed that *MAGE-C1/CT7* is involved in survival of malignant plasma cells, protecting myeloma cells against spontaneous as well as drug-induced (bortezomib-inhibitor of the 26S ubiquitin/proteasome) apoptosis. We also suggest that this CTA might play a role in cell cycle and speculate that silencing MAGE-C1/CT7 might represent a valuable therapeutic option for MM, in particular when applied in combination with proteasome inhibitors. However, the exact function of MAGE-C1/CT7 protein in the pathophysiology of MM is not yet understood [24].

## 4. Concluding Remarks

CTAs are attractive targets for immunotherapy in cancer due to its limited expression in somatic tissues. These antigens have been detected in MM patients and might be used for T-cell immunotherapy. However, we still know little about the actual role of CTAs in the biology of this incurable disease. Several studies demonstrate that the MAGE-C1/CT7 is commonly expressed in MM and has important role in the development and prognosis of the disease, making it a possible therapeutic target. Nevertheless, further *in vitro, in vivo* and clinical studies should be conducted in order to better understand the participation of MAGE-C1/CT7 and other CTAs in the MM tumorigenesis and to clarify the biological pathways in which these proteins act.
